# Combination of miR-99b-5p and Enzalutamide or Abiraterone Synergizes the Suppression of EMT-Mediated Metastasis in Prostate Cancer

**DOI:** 10.3390/cancers16101933

**Published:** 2024-05-19

**Authors:** Mohammad Waseem, Bi-Dar Wang

**Affiliations:** 1 Department of Pharmaceutical Sciences, School of Pharmacy and Health Professions, University of Maryland Eastern Shore, Princess Anne, MD 21853, USA; mwaseem@umes.edu; 2 Hormone Related Cancers Program, Greenebaum Comprehensive Cancer Center, University of Maryland, Baltimore, MD 21201, USA

**Keywords:** prostate cancer, miR-99b-5p, EMT, metastasis, CRPC, AA PCa, enzalutamide, abiraterone, PI3K/AKT/mTOR signaling, AR signaling

## Abstract

**Simple Summary:**

To date, effective treatment for metastatic prostate cancer (PCa) remains a clinical challenge. Our previous study demonstrated that tumor suppressive miR-99b-5p negatively regulates the expression of androgen receptor (AR) and mTOR, potentially serving as a therapeutic agent for aggressive PCa. In this study, we show for the first time that combination of miR-99b-5p mimic and second-generation AR antagonist (such as enzalutamide or abiraterone) simultaneously targets AR and mTOR signaling, consequently inhibiting epithelial-mesenchymal transition (EMT) in PCa. This novel combination therapy may provide a unique opportunity for effectively suppressing EMT-mediated metastasis in aggressive PC, especially African American PCa and castration-resistant prostate cancer (CRPC).

**Abstract:**

Prostate cancer (PCa) is the most frequently diagnosed cancer and second leading cause of cancer deaths among American men. Androgen deprivation therapy (ADT) has been systemically applied as a first-line therapy for PCa patients. Despite the initial responses, the majority of patients under ADT eventually experienced tumor progression to castration-resistant prostate cancer (CRPC), further leading to tumor metastasis to distant organs. Therefore, identifying the key molecular mechanisms underlying PCa progression remains crucial for the development of novel therapies for metastatic PCa. Previously, we identified that tumor-suppressive miR-99b-5p is frequently downregulated in aggressive African American (AA) PCa and European American (EA) CRPC, leading to upregulation of mTOR, androgen receptor (AR), and HIF-1α signaling. Given the fact that mTOR and HIF-1α signaling are critical upstream pathways that trigger the activation of epithelial–mesenchymal transition (EMT), we hypothesized that miR-99b-5p may play a critical functional role in regulating EMT-mediated PCa metastasis. To test this hypothesis, a series of cell biology, biochemical, and in vitro functional assays (wound healing, transwell migration, cell/ECM adhesion, and capillary-like tube formation assays) were performed to examine the effects of miR-99b-5p mimic on regulating EMT-mediated PCa metastasis processes. Our results have demonstrated that miR-99b-5p simultaneously targets MTOR and AR signaling, leading to upregulation of E-cadherin, downregulation of Snail/N-cadherin/Vimentin, and suppression of EMT-mediated PCa metastasis. MiR-99b-5p alone and in combination with enzalutamide or abiraterone significantly inhibits the EMT-mediated metastasis of AA PCa and EA CRPC.

## 1. Introduction

In the United States, PCa is the most commonly diagnosed cancer and second leading cause of cancer mortality (estimated with 299,010 new cases and 35,250 deaths in 2024) among American men [1]. Due to the high heterogeneity of PCa, the development of optimal treatment options for patients remains challenging because of the poor response towards therapies and tumor recurrence. Therefore, identification of novel drug targets is in urgent need for further developing individualized therapeutic strategies for PCa patients.

Androgen deprivation therapy (ADT) has been considered as a standard regimen for localized and advanced PCa patients. Despite an initial favorable response to ADT, most of the patients experienced disease recurrence within 18–24 months. Ultimately, the disease progresses to castration-resistant prostate cancer (CRPC), a metastatic PCa with poor prognosis [2,3]. Previous studies have demonstrated that PCa cells exhibit high plasticity (termed ‘lineage plasticity’), allowing PCa cells to adapt to ADT through cellular rewiring (i.e., via upregulation of AR, PI3K, or GATA2 signaling) and, therefore, develop drug resistance [4]. Second-generation hormone therapeutic agents, such as enzalutamide and abiraterone acetate, have been shown to improve overall survival and quality of life in metastatic CRPC patients [5,6,7,8,9,10]. Enzalutamide (Enz) is an androgen receptor (AR) antagonist that demonstrates potent efficacy against metastatic CRPC. Mechanistically, Enz blocks the translocation of the AR from the cytoplasm to the nucleus and prevents the binding of AR with its coregulators and chromatin binding sites, subsequently inhibiting the transcription of AR-downstream genes [11,12]. Abiraterone acetate (Abi) has been utilized as a first-class, selective inhibitor of cytochrome P450 (CYP) 17, a crucial enzyme responsible for extragonadal and testicular androgen synthesis. Abi along with prednisone has also been shown to improve the survival of and reduce side-effects for metastatic CRPC patients [10,13]. Although both Enz and Abi demonstrated clinical benefits, the overall survival of Enz/Abi-treated patients is only modestly increased and the majority of responders develop resistance over time [14,15]. For example, most Enz-treated patients who have had drastic reduction in PSA levels eventually develop resistance, with increasing PSA and/or progression of bone metastasis [16]. To date, AR-V7 synthesis, upregulation of CYP17 and/or alteration in AR signaling axis, expression of glucocorticoid receptor (GR), upregulation of PI3K/AKT/MAPK and/or GATA2 signaling, altered tumor microenvironment, and deregulation of microRNAs (miRNAs) have been considered as possible mechanisms involved in the acquired resistance to Enz and Abi [4,17,18]. For instance, AR-V7 is a splice isoform lacking the ligand-binding domain (LBD) of AR [19]. Therefore, an increased level of AR-V7 has been associated with disease progression and treatment failure of Enz and Abi in CRPC [20,21]. Upregulation AR signaling, due to *AR* gene amplification; increased stability of AR; or mutations in *AR*, could also lead to Enz and Abi resistance [22,23,24]. Due to the clinical challenges observed in CRPC, novel therapeutics that overcome drug resistance are still required.

MicroRNAs (miRNAs) are small non-coding RNAs that epigenetically regulate cellular processes by regulating gene expression at the post-transcriptional level. MiRNAs are frequently deregulated in cancer development/progression and drug resistance; therefore, miRNAs have been implicated as potential biomarkers and treatment response modulators in various cancers, including PCa [25,26,27,28,29,30]. Previously, we identified dozens of reciprocal miRNAs/mRNA pairings and associated miRNA-mRNA regulatory networks in African American (AA) PCa disparities [31,32]. Among these miRNA–mRNA pairings, the downregulation of miR-99b-5p and upregulation of *MTOR* has been implicated as a critical epigenetic event that contributes to tumor aggressiveness in AA PCa and the progression of CRPC [33,34]. Furthermore, our studies have shown that miR-99b-5p simultaneously targets and inhibits the expression of *MTOR*, *AR*, and *SMARCD1* (encoding SMARCD1, an AR coregulator), consequently blocking the translocation of the mTOR/AR/SMARCD1 complex from the cytoplasm to the nucleus, inhibiting the expression of mTOR/AR target genes, reversing metabolic rewiring, and sensitizing AA PCa and CRPC to Enz [34].

Epithelial–mesenchymal transition (EMT) has been implicated as a critical step during the development of distance metastases from a primary tumor [35]. Specifically, EMT is regulated by transcription factors such as Snail, Slug, ZEB1/2, and Twist1/2 [36,37]. These EMT-transcriptional factors (EMT-TFs) then suppress the expression of E-cadherin (an epithelial marker) and activate the expression of N-cadherin, Vimentin, and fibronectin (mesenchymal markers). The activation of EMT results in reduced cell–cell adhesion and triggers cell detachment from the primary tumor, leading to tumor invasion and migration/metastasis [38,39]. PI3K/AKT/mTOR and hypoxia/HIF1α signaling, in collaboration with other signaling pathways, induces the expression of EMT-TF genes (i.e., *SNAIL*, *ZEB1/2*, and *TWIST1/2*) and activates EMT in cancers, including PCa [40,41]. Additionally, AR signaling has also been implicated in the activation of EMT in PCa [42,43]. Due to the central/unique role of the reciprocal miR-99b-5p/*MTOR* pairing (downregulated/upregulated in AA PCa) in coordinating PI3K/AKT/mTOR, HIF1α, and AR signaling [31,33,34], we hypothesized that the miR-99b-5p/mTOR/AR/SMARCD1 signaling axis may play a critical functional role in regulating EMT-mediated metastasis in AA PCa and CRPC. To test this hypothesis, we performed a series of functional and biochemical experiments to examine whether transfection/overexpression of miR-99b-5p mimic could inhibit the expression of EMT-TF (Snail) and mesenchymal markers (N-cadherin and Vimentin) and restore the expression of epithelial marker (E-cadherin), ultimately inhibiting the metastatic capacities of AA PCa and CRPC cells. In addition, miR-99b-5p mimic was used as a single agent and in combination with Enz or Abi to assess the inhibitory effects on EMT-mediated migration and angiogenesis capacities in aggressive AA PCa and CRPC cells.

## 2. Materials and Methods

### 2.1. Cell Culture Maintenance and Conditions

The human PCa cell lines LNCaP, 22Rv1, C4-2B, and MDA PCa 2b were used in the current study. LNCaP is an EA PCa cell line derived from lymph nodes [44]. C4-2B is a CRPC cell lines derived from EA PCa [45], 22Rv1 is a CRPC cell line derived from EA patient [46], while MDA PCa 2b is an AA PCa cell lines derived from bone metastasis [47]. All the PCa cell lines used in this study were purchased from American Type Culture Collection (ATCC, Manassas, VA, USA). RPMI-1640 (Gibco, Waltham, MA, USA) with 10% fetal bovine serum (FBS, Gibco, Waltham, MA, USA) used as the medium to grow LNCaP and 22Rv1 cells. DMEM (Gibco, Waltham, MA, USA) with 10% FBS was used to grow C4-2B cells; BRFF-HPC1 (Athena ES, Baltimore, MD, USA) with 20% FBS was employed to grow MDA PCa 2b cells.

Human umbilical vascular endothelial cells (HUVECs) were maintained at 37 °C with 5% CO_2_ in EBM-2 media, as per the manufacturer’s instructions. HUVEC cells, at passages of 2–3, were used for the experiments. Furthermore, HUVECs cultured on Matrigel were exposed to the condition medium (CM). The preparation of the CM is described as follows: 1.5 × 10^5^ cells/mL of PCa cells were grown in 6-well plates coated with collagen I for proper attachment in each well. After 3–4 days, the culture medium was removed and replaced with fresh media in each well. The PCa cells were then incubated for 2–3 days and, thereafter, the media was collected and centrifuged at 1000× *g* for 5 min. Finally, the obtained supernatants were saved and defined as the CM.

### 2.2. MicroRNA Transfection and Drug Treatment Schedule in EA and AA PCa Cell Lines

Subsequently, 3 × 10^5^ cells of LNCaP, 22Rv1, C4-2B, or MDA PCa 2b cells were seeded and grown in a well of the 6-well plate; then, the PCa cells were grown for 24 h. Thereafter, the PCa cells were transfected with either nonsense RNA or miR-99b-5p mimic (Ambion, Austin, TX, USA). The transfected PCa cells were then grown overnight and treated with vehicle, Enz, or Abi for an additional 48 h. The treatment groups were as follows: NC (transfection of nonsense control, and vehicle treatment), miR-99b-5p (transfection of miR-99b-5p mimic, and vehicle), Enz (transfection of nonsense RNA, and 20 μM of Enz), Abi (transfection of nonsense RNA and 10 μM Abi), miR-99b-5p/Enz (combination of miR-99b-5p mimic and 20 μM of Enz), and miR-99b-5p/Abi (combination of miR-99b-5p mimic and 10 μM of Abi). All the cells were grown in 5% CO_2_ incubator at 37 °C. A total of 20 μM of Enz and 10 μM of Abi were the concentrations used in the experiments, according to previous studies [34,48].

### 2.3. Immunofluorescence Staining for PCa Cell Models

In brief, 4 × 10^4^ cells were grown on a cover slip for 24 h in 5% CO_2_ incubator at 37 °C. Thereafter, cells were washed with 1 × PBS, fixed in 4% paraformaldehyde, and permeabilized with 0.1% Triton X-100. The fixed and permeabilized PCa cells were blocked for 1 h with 2% BSA in 1 × PBS. After blocking, primary antibody was applied to label the protein of interest. After incubating the sample with primary antibody overnight at 4 °C, the cells were washed twice with 1×PBS then incubated with secondly antibody conjugated with fluorescence for 1 h at room temperature. Finally, the cells were mounted with DAPI-containing mounting medium (Invitrogen, Waltham, MA, USA). The antibodies used in the study are listed as follows: N-cadherin (catalog# 13116, 1:200 dilution, Cell Signaling Technology, Danvers, MA, USA; or catalog# sc-59987, 1:100 dilution, Santa Cruz Biotechnology, Dallas, TX, USA), E-cadherin (catalog# 3195, 1:200 dilution, Cell Signaling Technology, Danvers, MA, USA; or catalog# sc-8426, 1:100 dilution, Santa Cruz Biotechnology, Dallas, TX, USA), Vimentin (catalog# 5741, Cell signaling, 1:200 dilution, Danvers, MA, USA), Snail (catalog# 3879, 1:200 dilution, Cell Signaling, 1:100 dilution, Danvers, MA, USA; or catalog# sc-271977, Santa Cruz Biotechnology, Dallas, TX, USA), and Alexa-Fluor-488-conjugated anti-rabbit and Alexa-Fluor-594-conjugated anti-mouse antibodies (catalog# A32731 and #A32744, 1:500 dilution, respectively, from Invitrogen, Waltham, MA, USA). The immunofluorescence-stained cells were visualized and the images were captured using Olympus fluorescence microscopy (Waltham, MA, USA). The images of the immunofluorescence signals were captured from 3–4 random areas, and image analysis was performed using CellSens V1.18 software (Olympus, Waltham, MA, USA).

### 2.4. Western Blot Analysis

Western blot assays were performed using the protocol as previously described [33,34,49]. The PCa cells were collected, and total proteins were extracted using M-PER with a protease and phosphatase inhibitor cocktail (Thermo Fisher Scientific, Waltham, MA, USA). Quantification of total protein concentrations were performed using the BCA assay kit (Thermo Fisher Scientific, Waltham, MA, USA). The protein samples were loaded in Bolt 4–12% Bis-Tris mini protein gels (Thermo Fisher Scientific, Waltham, MA, USA) for electrophoresis. The primary antibodies used in the study were as follows: antibodies for E-cadherin (catalog# 3195, 1:1000 dilution), N-cadherin (catalog# 13116, 1:1000 dilution), Vimentin (catalog# 5741, 1:1000 dilution), Snail-1 (catalog# 3879, 1:1000 dilution), and β-actin (catalog# 4970S, 1:2000 dilution) from Cell Signaling Technology (Danvers, MA, USA). The secondary antibodies were anti-rabbit IgG-HRP (catalog# 4030-05, 1:10000 dilution) and anti-mouse IgG-HRP (catalog# 1033-05, 1:5000 dilution) antibodies from Thermo Fisher Scientific (Waltham, MA, USA).

### 2.5. Evaluation of Tumor Migration by Using Wound Healing Assay

PCa cells were cultured until 95–100% confluence in a 6-well plate. An elongated scratch was created using a pipette tip; the floating cells were then removed and the remaining attached cells were washed with 1 × PBS. The cells then underwent treatments of NC, miR-99b-5p mimic, Enz, Abi, miR-99b-5p/Enz, and miR-99b-5p/Abi, as described above. To visualize the migration capacities of the PCa cells with different treatments, cell migrations of all groups were observed and photographed using an inverted phase contrast microscopic system (Olympus, Waltham, MA, USA) at 0 and 48 h. The migration capacities were measured by calculating the migrating distance of the cells between 0 and 48 h. The migrating distance (scratch width at 0 h–scratch width at 48 h) of the NC-treated cells was defined as 100%. Scratch widths at 0 and 48 h were defined as the distances between the two yellow dashed lines at 0 h and 48 h, respectively. The data were generated from 3 independent scratch wound healing assays per treatment group. The statistics and bar graphs were performed and plotted using Prism 9 program (GraphPad Software, La Jolla, CA, USA).

### 2.6. Transwell Migration Assay

A migration assay was performed in 12-well transwells with a pore size of 4.0 µM (Corning, Wilkes Barre, PA, USA). The PCa cells were first grown for 24 h and then the cells were transfected with either nonsense RNA or miR-99b-5p mimic. Second, 100 µL of transfected PCa cells (1 × 10^4^) suspended in serum-reduced medium were transferred to the upper chambers of the transwell compartment. A total of 600 µL of medium containing 10% FBS as a chemoattractant was added to the lower chamber of the transwell compartment. Third, the transferred PCa cells were then treated with either vehicle, enzalutamide, or abiraterone for an additional 48 h and subjected to staining of the migrated cells. Specifically, non-migrated cells (cells remaining on the top side of the membrane in the insert) were wiped off using a cotton swab. The migrated cells (cells migrated to the bottom side of the membrane in the insert) were fixed with 100% methanol (Sigma, St. Louis, MO, USA) and stained with 0.5% crystal violet (Sigma, St. Louis, MO, USA) for counting the number of migrated cells to determine the migration capacities of the PCa cells. The cells that migrated to the bottom surface were visualized and counted in 3–4 randomly chosen areas using an inverted microscope (Olympus, Waltham, MA, USA) at 10× magnification. The data were obtained from 3–4 independent experiments. The migrated cells (%) were determined via normalization of the migrated cells in the experimental group to the migrated cells in the NC group. The data were analyzed using the Prism 9 program (GraphPad Software, La Jolla, CA, USA) for graphing and statistical analysis.

### 2.7. Cell Adhesion Assay

For this procedure, 1 × 10^5^ of PCa cells were seeded in a 24-well plate coated with 35 µg/mL of collagen and 10 µg/mL fibronectin. Cells were cultured for 24 h before transfection with nonsense RNA or miR-99b-5p mimic. Then, the transfected PCa cells were further treated with vehicle, Enz, or Abi. After treatment for 48 h, the medium was aspirated and the wells were washed thrice with 1 × PBS. Furthermore, the cells were then fixed with 100% chilled methanol for 15 min and stained using 0.5% crystal violet for 20 min. Overstained cells were washed 3–4 times with 1 × PBS to remove excess dye. The number of adherent cells (stained by crystal violet) was visualized and counted using an inverted phase-contrast microscopic system (Olympus, Waltham, MA, USA). The number of adherent cells (averaged from *n* = 3–4) in the NC group was defined as 100%, and was used to normalize the adherent cells (%) for other experimental groups. The data analyses were achieved using the Prism 9 program (GraphPad Software, La Jolla, CA, USA) for graphing and statistical analysis.

### 2.8. Angiogenesis (Tube Formation) Assay

A total of 70 µL of Matrigel was placed into each well of a 96-well plate and allowed to polymerize for 1 h at 37 °C. HUVEC cells at a passage of 2–3 were then co-cultured with 100 μL of PCa-derived CM. After 24 h, tubular morphology of HUVECs was observed and images were captured using an inverted phase contrast microscopic system optical microscope (Olympus, Waltham, MA, USA) with a 20× objective lens. All observations were performed 3–4 times, and each experiment was repeated 3–4 times.

### 2.9. Statistical Analysis

All the data were calculated and are presented as mean ± standard deviation (SD). The multiple comparisons were analyzed using analysis of variance (ANOVA) with Tukey’s post hoc test. The statistics (i.e., significance based on *p*-values, etc.) were performed using GraphPad Prism 9.0 (Graph Pad Software, La Jolla, CA, USA).

## 3. Results

### 3.1. Overexpression of miR-99b-5p Mimic and Enz/Abi Treatment Modulate the Expression Levels of N Cadherin, E-cadherin, Vimentin, and Snail in EA and AA PCa Cells

Four cell lines that were derived from European American (EA) PCa and AA PCa were used in our study. LNCaP is a EA cell line that was derived from a metastatic lymph node lesion of PCa that was AR positive, exhibiting androgen-sensitive tumor growth [44]. C4-2B was derived from a bone metastasis that was established in nude mice after injecting LNCaP-derived, castration-resistant C4-2 cells [45]. 22Rv1 is an EA PCa cell line derived from a xenograft serially propagated in mice after castration-induced regression and relapse of the parental CWR22 xenograft [46]. MDA PCa 2b is a PCa cell line derived from androgen-refractory bone metastasis of an AA PCa patient. MDa PCa 2b cell line was shown to express similar AR and PSA levels as compared to the clinical samples [47]. In summary, LNCaP was used as an AR-positive, androgen-sensitive EA PCa model. C4-2B and 22Rv1 were served as AR-positive EA CRPC models, while MDA PCa 2b was used as an AR-positive, androgen-independent AA PCa model.

**Figure 1:**
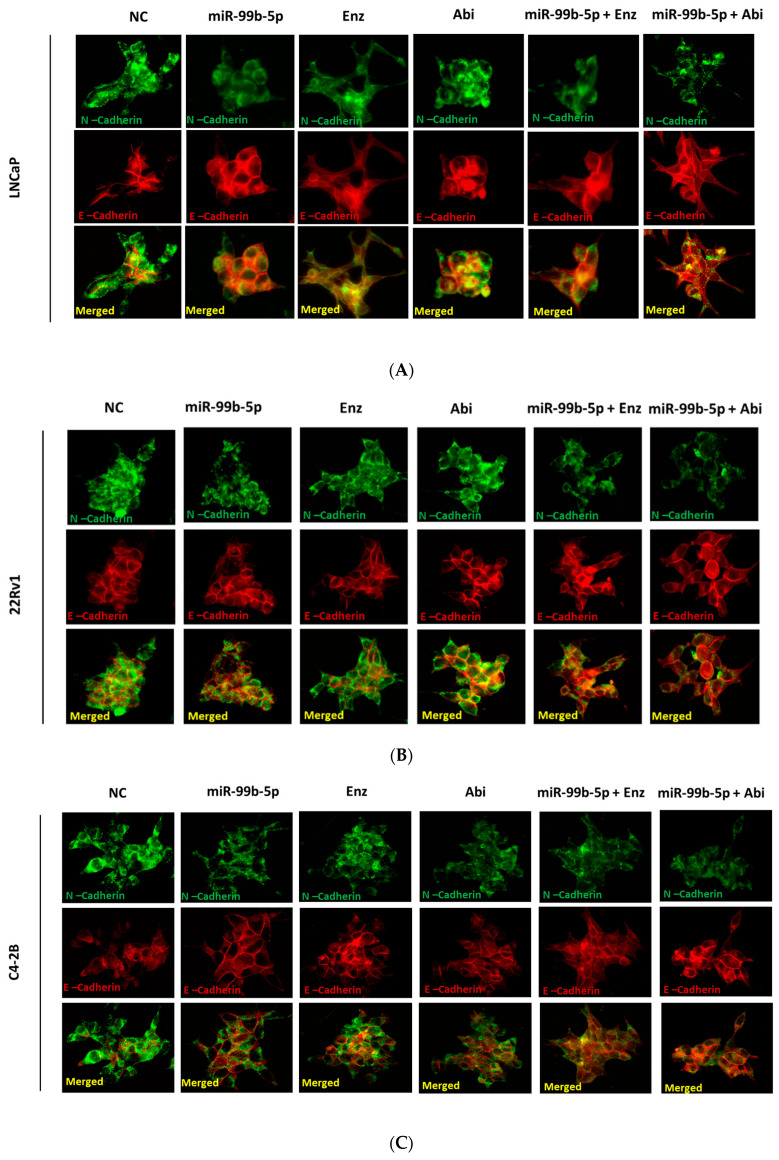

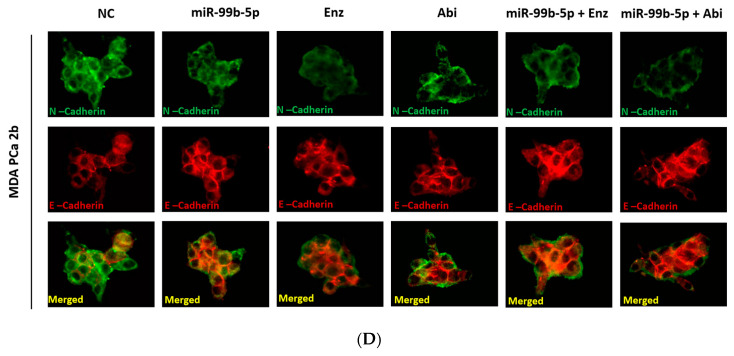
Immunofluorescence revealed an opposite effect of miR-99b-5p mimic and/or Enz/Abi on regulating E-cadherin and N-Cadherin expression in AA PCa and EA PCa cells. Immunofluorescence assays were performed to examine the protein levels of E-Cadherin (red fluorescence) and N-Cadherin (green fluorescence) signals in (**A**) LNCaP, (**B**) 22Rv1, (**C**) C4-2B, and (**D**) MDA PCa 2b cells treated with NC, miR-99b-5p mimic, Enz, Abi, miR-99b-5p/Enz, and miR-99b-5p/Abi. Merged images were obtained by overlaying E-Cadherin and N-Cadherin signals to reveal the subcellular localization of these two proteins (i.e., yellow signal indicates the colocalization of E-Cadherin and N-Cadherin in cytoplasm). These images are presented as representative fluorescence staining images from 3–4 independent experiments.

To assess the effects of miR-99b-5p and Enz/Abi on EMT-mediated metastasis, the expression levels of EMT markers E-cadherin (epithelial), Snail (EMT-TF), N-cadherin and Vimentin (mesenchymal), and Snail were examined using immunofluorescence in EA PCa (LNCaP, 22Rv1, and C4-2B) and AA PCa (MDA PCa 2b) cells transfected with nonsense RNA or miR-99b-5p mimic in the absence/presence of Enz or Abi. As shown in Figure 1, overexpression of miR-99b-5p mimic resulted in a generalized reduction in N-cadherin (green fluorescence, top panels) and increase in E-cadherin (red fluorescence, middle panels) protein levels, when comparing miR-99b-5p mimic-treated to NC-treated EA and AA PCa cells. Similarly, either Enz or Abi treatment resulted in the reduction of N-cadherin signals in EA and AA PCa cell lines, when compared to the NC controls (Figure 1A–D). In contrast, an enhanced level of E-cadherin signals was observed in all EA and AA PCa cell lines treated with Enz (or Abi) vs. NC. In addition, a significant reduction in N-cadherin and significant increase in E-cadherin signals were detected in EA and AA PCa cells treated with miR-99b-5p/Enz or miR-99b-5p/Abi combination vs. NC control. Notably, miR-99b-5p/Enz or miR-99b-5p/Abi combination caused lower N-cadherin and higher E-cadherin expression levels compared to miR-99b-5p or Enz/Abi as single agent. Taken together, our results suggested that miR-99b-5p or Enz/Abi effectively inhibit EMT by inhibiting mesenchymal marker N-cadherin and restoring the epithelial marker E-cadherin. Moreover, the combination of miR-99b-5p mimic with Enz or Abi resulted in additive inhibition of EMT, as compared to miR-99b-5p mimic or Enz/Abi alone.

**Figure 2:**
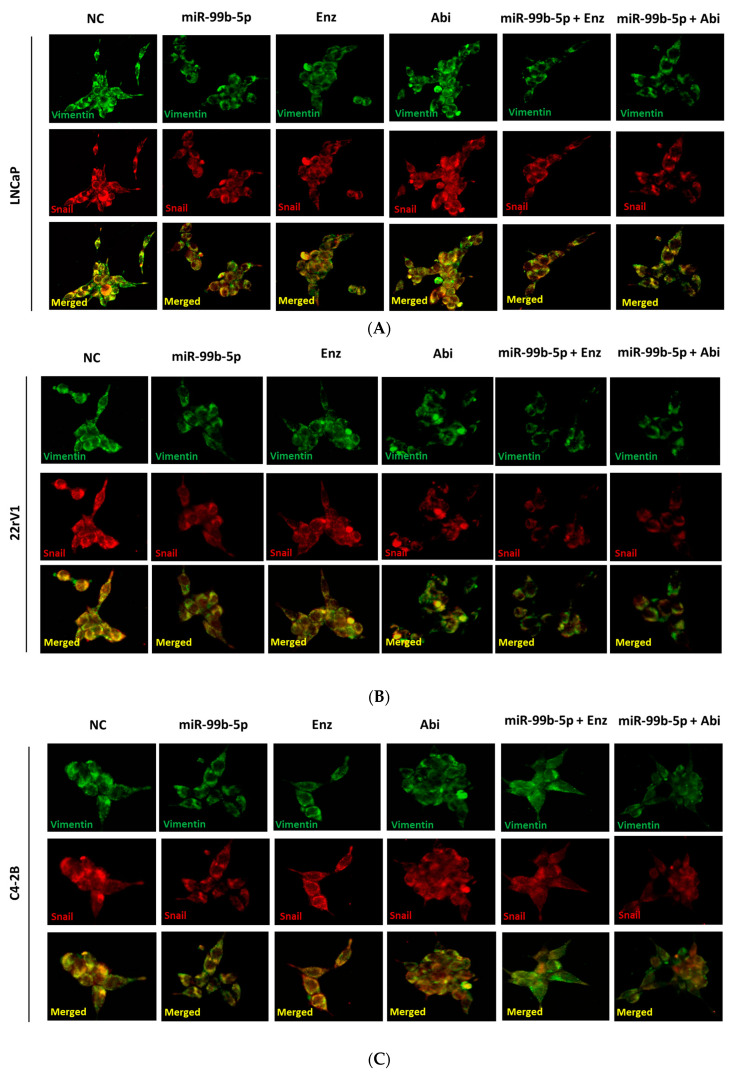

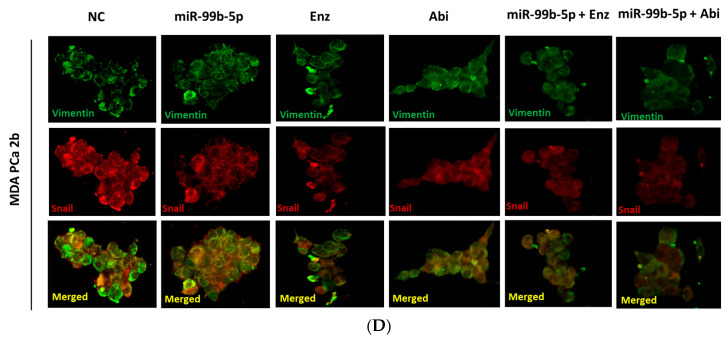
Immunofluorescence staining assays of Vimentin and Snail proteins in AA and EA PCa cells under different treatments. Immunofluorescence assays were performed to examine the protein levels of Snail (red fluorescence) and Vimentin (green fluorescence) signals in (**A**) LNCaP, (**B**) 22Rv1, (**C**) C4-2B, and (**D**) MDA PCa 2b cells treated with NC, miR-99b-5p mimic, Enz, Abi, miR-99b-5p/Enz, and miR-99b-5p/Abi. Merged images were obtained by overlaying E-Cadherin and N-Cadherin signals to reveal the subcellular localization of these two proteins (i.e., yellow signals indicate the colocalization of Snail and Vimentin proteins in cytoplasm). These images are presented as representative fluorescence staining images from 3–4 independent experiments.

As shown Figure 2A–C, overexpression of miR-99b-5p mimic caused a reduction in both Vimentin (green fluorescence) and Snail (red fluorescence) levels, compared to the NC treatment in all EA PCa cells. In AA PCa (MDA PCa 2b) cells, transfection of miR-99b-5p caused the inhibition of Snail protein level, but did not significantly change Vimentin expression, as compared to its NC treatment (Figure 1D, middle panels). Similarly, Enz or Abi treatment resulted in a reduction in Snail in all EA and AA PCa cell lines, as compared to the NC treatment (Figure 2A–D, Snail panels). Compared to NC treatment, Enz or Abi treatment caused a reduction in Vimentin protein levels in all EA PCa cells but not AA PCa cells (Figure 2A–D, Vimentin panels). Notably, miR-99b-5p/Enz or miR-99b-5p/Abi treatment further downregulated Snail and Vimentin in all three EA PCas, compared to either miR-99b-5p or Enz/Abi as single agents (Figure 2A–C). In AA PCa, Snail (but not Vimentin) expression levels were significantly inhibited in miR-99b-5p/Enz and miR-99b-5p/Abi combination vs. single agents of miR-99b-5p, Enz, or Abi (Figure 2D).

### 3.2. Immunoblotting Validation of N-Cadherin, E-Cadherin, Vimentin, and Snail Protein Levels of EA and AA PCa Cells in Response to miR-99b-5p Mimic, Enz, Abi, miR-99b-5p/Enz, and miR-99b-5p/Abi

To verify the immunofluorescence assay results (Figure 1 and Figure 2), Western blot analyses of the EMT markers were performed using cell lysates from EA PCa (LNCaP, 22Rv1, and C4-2B) and AA PCa (MDA PCa 2b) cells treated with NC, miR-99b-5p mimic, Enz, Abi, miR-99b-5p/Enz, and miR-99b-5p/Abi, respectively.

**Figure 3:**
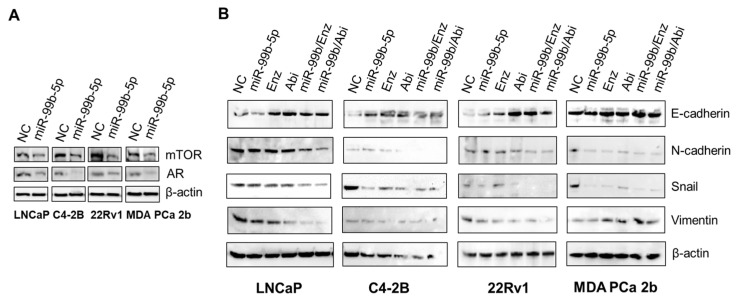
Western blot analyses of the EMT markers E-cadherin, N-cadherin, Snail, and Vimentin proteins in EA and AA PCa cells under different treatments. (**A**) Western blot analysis of mTOR and AR levels upon transfection of miR-99b-5p mimic in EA and AA PCa cells. (**B**) Western blot analyses of N-cadherin, E-cadherin, Snail, and Vimentin proteins in PCa cells under treatments of NC, miR-99b-5p, Enz, Abi, miR-99b-5p/Enz, and miR-99b-5p/Abi. β-actin was used as an endogenous control. The Western blot images are representative images selected from 3 independent Western blot experiments. The uncropped blots are shown in the Supplementary Materials.

First, the cell lysates from all cell lines transfected with NC and miR-99b-5p mimic were subjected to Western blot for examining mTOR and AR protein levels. As shown in Figure 3A, overexpression of miR-99b-5p resulted in downregulation of mTOR and AR in all EA and AA PCa cell lines, confirming that *MTOR* and *AR* were targeted and inhibited by miR-99b-5p, as previously described [33,34]. Second, the cell lysates from EA and AA PCa under all six treatments were collected and subjected to examine the protein expression levels of E-cadherin (epithelial), Snail (EMT-TF), N-cadherin, and Vimentin (mesenchymal). As shown in Figure 3B, E-cadherin was upregulated, while Snail and N-cadherin were downregulated upon treatments of miR-99b-5p mimic, Enz, or Abi. Similar to the immunofluorescence staining results, an additive inhibitory effect of Snail and N-cadherin and additive upregulated effect of E-cadherin was observed in all EA and AA PCa cells under combined treatment (miR-99b-5p/Enz or miR-99b/Abi) vs. single agent (miR-99b-5p, Enz, or Abi alone). Vimentin (mesenchymal marker) was also downregulated upon miR-99b-5p mimic, Enz, Abi, miR-99b-5p/Enz, or miR-99b-5p/Abi in EA PCa cell lines (but not in AA PCa cell line, MDA PCa 2b) (Figure 3B).

These results are consistent with the immunofluorescence assay results, suggesting that miR-99b-5p, Enz, and Abi may function as single agents and synergize in combination to inhibit activation of EMT in EA and AA PCa cells.

### 3.3. Wound Healing Assays Revealed That miR-99b-5p Mimic and Enz/Abi Treatment Suppress the Migration of EA and AA PCa Cell Models

To further test whether inhibition of EMT by miR-99b-5p/Enz/Abi affects the cell migration of EA and AA PCa, we performed scratch assays to examine migration capacities/mobilities of EA and AA PCa cells treated with miR-99b-5p, Enz, Abi, miR-99b-5p/Enz, and miR-99b-5p/Abi. Specifically, the migration rates of the EA and AA PCa (under different treatments) were determined in a window of 48 h.

**Figure 4:**
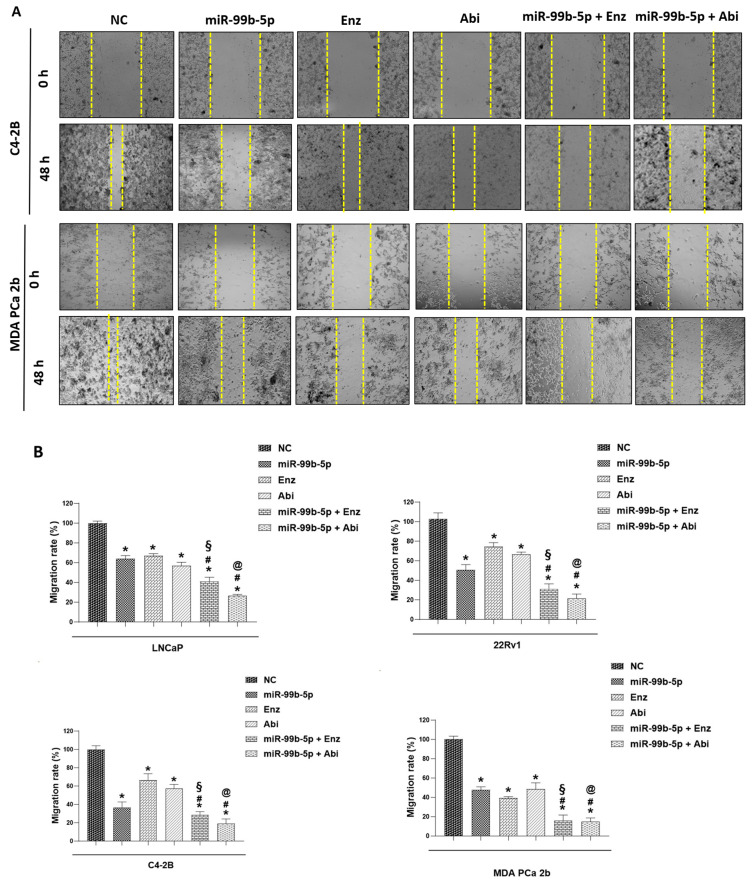
Wound-healing assays to determine migration of EA PCa cell lines (LNCaP, 22Rv1, and C4-2B) and AA PCa cell lines (MDA PCa 2b) transfected with NS or miR-99b-5p mimic in the absence or presence of 20 µM Enz or 10 µM Abi. (**A**) Representative phase-contrast microscope images showing the areas covered by the PCa cells (with different treatments) at 0 and 48 h. (**B**) Bar graphs showing migration rates of the PCa cells (under different treatments) within 48 h. The migration rate was determined within 48 h using a wound-healing assay (described in Materials and Methods). Significantly different migration rates were determined: * *p*-value < 0.05 in either treatment vs. NC group, ^#^ *p*-value < 0.05 in miR-99b-5p/Enz (or miR-99b/Abi) vs. miR-99b-5p treated cells, ^§^ *p*-value < 0.05 in miR-99b-5p/Enz vs. Enz-treated cells, and ^@^ *p*-value < 0.05 in miR-99b-5p/Abi vs. Abi-treated cells. The migration rate of the NC treated cells was defined as 100% for data normalization across different treatment groups, and *p*-values were determined using ANOVA with Tukey’s post hoc test. Each value was derived from the mean ± SD (*n* = 3–4).

Compared to the NC-treated cells, miR-99b-5p-, Enz-, or Abi-treated cells resulted in a generalized lower migration capacity (with wider spaces between the migrating tumor cells at 48 h, defined by the two yellow dashed lines) of EA and AA PCa cells. Additionally, the treatment combination (miR-99b-5p/Enz or miR-99b-5p/Abi) synergizes the inhibitory effects on tumor migrations in all EA and AA PCa cell lines (Figure 4A). By calculating the migration distance per 48 h (defined as migration rate in this study), bar graphs showing the migration rates of the PCa cells in all treatments are presented in Figure 4B. Compared to the NC treated cells (migration rate was defined as 100%), mi-99b-5p treatment caused a generalized reduction (with 40–60% decrease) in migration rates. Enz and Abi treatments resulted in a 35–55% reduction in migration rates in LNCaP (androgen-sensitive EA PCa) and MDA PCa 2b (AA PCa) and a slighter suppression (with 25–30% decrease) of migration rates in EA CRPC (22Rv1 and C4-2B) cells. Notably, the combination of miR-99b-5p mimic with either Enz or Abi synergistically inhibits migration rates (with a 60–85% decrease in migration rates) in all the EA and AA PCa cells. These results strongly suggested that miR-99b-5p, Enz, and Abi can individually and synergistically inhibit tumor migration as single agents and in combinations (likely through the suppression of EMT activation).

### 3.4. Overexpression of miR-99b-5p and Enz/Abi Negatively Regulates EMT-Mediated Migration in PCa Cell Lines Based on Transwell Assays

**Figure 5:**
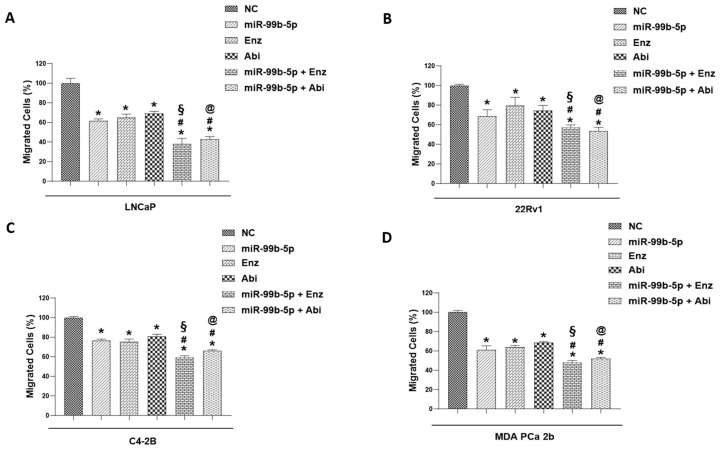
Transwell-mediated migration assays of the PCa cells treated with NC, miR-99b-5p mimic, Enz, Abi, miR-99b-5p/Enz, or miR-99b-5p/Abi. Bar graph showing the migration capacities of NC-, miR-99b-5p-, Enz-, Abi-, miR-99b-5p/Enz-, and miR-99b-5p/Abi-treated LNCaP (**A**), 22Rv1 (**B**), C4-2B (**C**), and MDA PCa 2b (**D**) cells. Note the migration capacities were defined by % of migrated cells through transwell assays. Significantly different migration capacities (defined as % migrated cells) were determined: * *p*-value < 0.05 in either treatment vs. NC group, ^#^ *p*-value < 0.05 in miR-99b-5p/Enz (or miR-99b/Abi) vs. miR-99b-5p treated cells, ^§^ *p*-value < 0.05 in miR-99b-5p/Enz vs. Enz-treated cells, and ^@^ *p*-value < 0.05 in miR-99b-5p/Abi vs. Abi-treated cells. The migration rate of the NC-treated cells were defined as 100% for data normalization across different treatment groups, and *p*-values were determined based on ANOVA with Tukey’s post hoc test. Each value was represented as the mean ± SD (*n* = 3).

It is evident that tumor dissemination initiates with tumor invasion through the basement membranes, followed by migration to surrounding tissues, intravasation into blood vessels, and finally achieving tumor migration and colonization at distant organ sites [50]. The transwell assay, an in vitro assay mimicking the migration process, was utilized to evaluate the tumor migration capacities of PCa cells under miR-99b-5p and/or Enz/Abi treatments. Specifically, the efficacies of miR-99b-5p, Enz, and Abi (as single agents or in combinations) on inhibiting the migration capacities of LNCaP, 22Rv1, C4-2B, and MDA PCa 2b cells were assessed using transwell assays (Figure 5). As shown in Figure 5A–D, miR-99b-5p mimic, Enz, and Abi as single agents resulted in a generalized reduction (25–40%, 20–35%, and 20–30% decrease, respectively) in migration capacities when compared to NC treatments in EA and AA PCa cells. On the other hand, combination of miR-99b-5p with Enz further suppressed tumor migration capacities when compared to miR-99b mimic (with an additional 15–25% decrease) or Enz alone (with an additional 20–30% decrease) in EA and AA PCa cells. Similarly, a combination of miR-99b-5p mimic with Abi further suppressed tumor migration by an additional 10–20% and 10–30% decrease in migration capacities, when compared to miR-99b-5p mimic or Abi alone, respectively, in EA and Aa PCa cells (Figure 5A–D). Taken together, the cell migration assay results have suggested that miR-99b-5p mimic, Enz, and Abi (as single agents or in combination) suppress EMT-mediated cell migration of EA and AA PCa cells.

### 3.5. Treatment of miR-99b-5p Mimic or Enz/Abi Reduces Cancer Cell Adhesion of PCa Cells

Cell adhesion to the extracellular matrix (ECM) is one of the critical steps promoting tumor growth, invasion, and metastasis [51]. To test how miR-99b-5p and Enz/Abi treatments affect tumor cell adhesion to ECM, a series of cell adhesion assays were conducted in EA and AA PCa cells treated with NC, miR-99b-5p, Enz, Abi, miR-99b-5p/Enz, and miR-99b-5p/Abi. Specifically, PCa cells transfected with nonsense RNA or miR-99b-5p mimic were grown on the ECM-coated plate, and were then treated with vehicle, Enz, or Abi. The assays allowed us to visualize the efficiencies of PCa cell adherence on ECM in response to different treatments.

**Figure 6:**
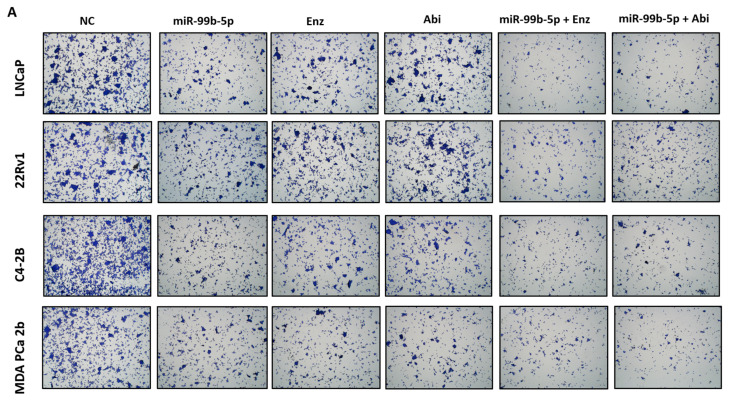

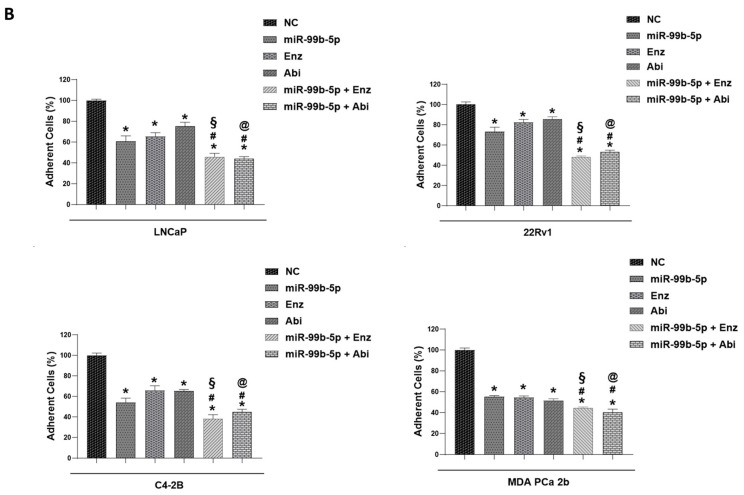
Cell adhesion assays of the PCa cells under treatments of NC, miR-99b-5p mimic, Enz, Abi, miR-99b-5p/Enz, and miR-99b-5p/Abi. (**A**) Representative images showing adhesion capacities of EA and AA PCa cells to extracellular matrix (ECM) in response to treatments of NC, miR-99b-5p mimic, Enz, Abi, miR-99b-5p/Enz, and miR-99b-5p/Abi. (**B**) Bar graphs quantifying the cell adhesion capacities (to ECM) of the PCa cells under different treatments. Significantly different cell-EMC adhesion capacities were determined: * *p*-value < 0.05 in either treatment vs. NC group, ^#^ *p*-value < 0.05 in miR-99b-5p/Enz (or miR-99b/Abi) vs. miR-99b-5p treated cells, ^§^ *p*-value < 0.05 in miR-99b-5p/Enz vs. Enz-treated cells, and ^@^ *p*-value < 0.05 in miR-99b-5p/Abi vs. Abi-treated cells. The migration rate of the NC treated cells was defined as 100% for data normalization across different treatment groups, and *p*-values were measured using ANOVA with Tukey’s post hoc test. Each data value was taken from the mean ± SD (*n* = 3).

As shown in Figure 6A, we observed that transfection of miR-99b-5p resulted in a reduction in cell adhesion capacities (with a 25–45% decrease, Figure 6B) in EA and AA PCa cells, as compared to NC. On the other hand, Enz and Abi treatments resulted in a generalized reduction (with a 20–50% decrease) in cell adhesion capacities of the EA and AA PCa cells, when compared to the NC groups. Similar to the migration and cell adhesion assays, the combined treatment of miR-99b-5p/Enz or miR—9b-5p/Abi induced a synergistic inhibition (with a 50–65% reduction) of cell adhesion capacities in the EA and AA PCa cell models, when compared to either miR-99b-5p, Enz, or Abi alone. Taken together, these results suggest that treatment of miR-99b-5p, Enz, and Abi as single agents or in combinations may individually or synergistically disrupt PCa cell adhesion to ECM.

### 3.6. Treatment of miR-99b-5p Mimic and Enz/Abi Modulates Angiogenesis Process in PCa Cells

HUVEC is an endothelial cell line with an ability to form capillary-like projections under in vitro conditions. It has been shown that culturing HUVEC cells with ECM components facilitates the development of a cellular branched structure and tubule-like projections, mimicking the in vivo process of angiogenesis [52,53].

**Figure 7:**
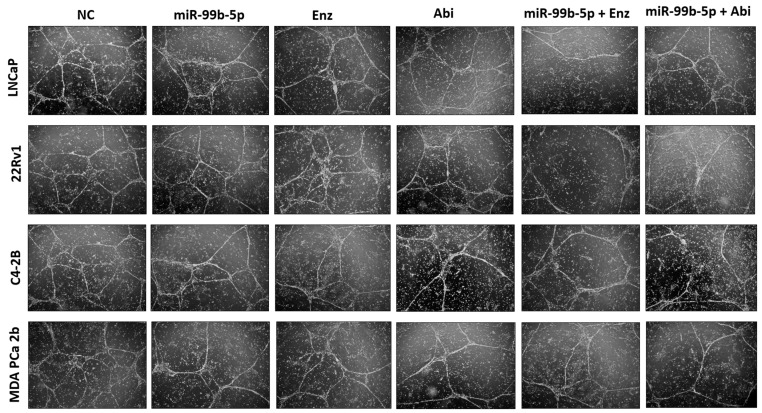
Representative phase-contrast microscope images showing the capillary-like tube formation of HUVEC cells incubated with condition medium derived from PCa cells under different treatments. The images were captured using an inverted phase-contrast microscope, and the representative images were selected from 3–4 areas of at least two independent tube formation assays per cell treatment group.

In this study, tubule formation efficiency was employed as an index to evaluate the angiogenesis capacities of the PCa cells under different treatments. First, HUVEC cells were exposed to the condition medium (CM) derived from PCa cells treated with NC, miR-99b-5p, Enz, and Abi and then they were subjected to tube formation assays. As shown in Figure 7, HUVEC cells successfully developed tubule-like structures after being incubated with CM derived from NC-treated PCa cells. In contrast, miR-99b-5p mimic, Enz, and Abi as single agents caused a slight to moderate reduction in tube formation in all PCa cells when compared to the NC groups. Moreover, miR-99b-5p/Enz and miR-99b-5p/Abi combinations resulted in a more significant reduction in tube formation, as compared to the single agent treatment with miR-99b-5p mimic, Enz, or Abi alone (Figure 7). These results have implicated that miR-99b-5p, Enz, and Abi may inhibit angiogenesis, and miR-99b-5p/Enz and miR-99b-5p/Abi combinations further synergize the inhibition of the angiogenesis process in PCa.

## 4. Discussion

EMT is a molecular cellular program that is required for cell development, wound healing, fibrosis, and cancer progression/metastasis [54,55]. Previous studies have also highlighted the critical role of EMT plasticity in PCa metastasis and treatment resistance [56,57]. Several signaling pathways have been identified as upstream regulators for activation of EMT in cancers, including Wnt, Notch, TGFβ, PI3K/AKT/mTOR, JAK/STAT, and hypoxia/HIF-1α signaling pathways [40,56]. These cell-intrinsic signaling pathways cooperate to induce the transcriptional activation of EMT-TFs, such as SNAIL, ZEB1/2, and Twist1/2, subsequently triggering the EMT process for induction of the transition to the mesenchymal state of the tumor cells [40,56,58].

Numerous studies have revealed that miRNAs function in promoting or inhibiting PCa metastasis [59,60]. For instance, miR-9, mR-21, and miR-181a have been shown to promote EMT, while miR-34, miR-130b, miR-200b, miR-204, and miR-573 have been involved in suppressing EMT [59,60]. Our previous studies have highlighted the deregulated miRNA–mRNA interaction as one of the critical epigenomic factors regulating PCa aggressiveness and treatment resistance [31,32]. Among the identified reciprocal miRNA/mRNA pairings involved in aggressive PCa, miR-99b-5p/*MTOR* (upregulated/downregulated) pairing has been revealed as a central miRNA/mRNA pairing coordinating PI3K/AKT/mTOR signaling with HIF-1α and VEGF pathways [33,34]. TGFβ collaborates with PI3K/AKT signaling to activate mTOR and NF-κB, activating the expression of EMT-TF genes. A previous study has also shown that mTORC1 and mTORC2 promote EMT-mediated metastasis through activation of RhoA and Rac1 in colorectal cancer [61].

In this study, restoring miR-99b-5p (which is downregulated in AA PCa and CRPC [31,33,34]) resulted in downregulation of mTOR, theoretically inhibiting the mTOR/NF-κB-mediated expression of *SNAIL1/2*, *ZEB1/2*, and *TWIST1/2*. This hypothesis was validated by our immunofluorescence staining and Western blot assays of Snail protein levels in NC vs. miR-99b-5p mimic-treated PCa cells (Figure 2 and Figure 3). Snail is an EMT-TF that negatively regulates the expression of E-cadherin [40,58]. Theoretically, overexpression of miR-99b-5p (in this study) would cause inhibition of mTOR-mediated activation of Snail, thereby leading to reactivation of E-cadherin, reversing the mesenchymal state to the epithelial state, and inhibiting metastasis of PCa. The upregulation of E-cadherin (epithelial marker); downregulation of Snail (EMT-TF, and downstream gene of mTOR), Vimentin, and N-cadherin (mesenchymal markers); and reduction of migration capacity successfully validates the suppressive role of miR-99b-5p in regulating EMT and EMT-mediated metastasis in PCa. Downregulation of miR-99b-5p and upregulation of mTOR has also been shown to activate HIF-1α and VEGF signaling in aggressive PCa [31]. Although the role of hypoxia/HIF-1α signaling in EMT remains unclear in PCa, HIF-1α signaling has been implicated to induce EMT in other cancers [62,63,64]. VEGF is one of components secreted by cancer-associated fibroblasts (CAFs) that promotes EMT in cancer [40]. In addition, HIF-1α-mediated activation of VEGF-A has been found to promote the induction of EMT [65]. Thus, downregulation of miR-99b-5p is likely to activate HIF-1α and VEGF signaling (via mTOR) and upregulation of VEGF-A (a miR-99b-5p target), subsequently leading to the induction of EMT in AA PCa and EA CRPC. Together, it explains why miR-99b-5p expression results in downregulation of EMT markers (Figure 1, Figure 2 and Figure 3), inhibition of metastasis (Figure 4, Figure 5 and Figure 6), and angiogenesis (Figure 7) in PCa cells.

Androgen signaling is another pathway implicated in the regulation of EMT in PCa. Activation of the androgen receptor (AR) has been shown to inhibit E-cadherin expression and promote activation of EMT [42,43], and androgen-mediated β-catenin signaling contributes to the induction of EMT in PCa [66]. However, several studies have also implicated that ADT itself may contribute to the development of EMT, leading to PCa invasion/metastasis and CRPC progression/metastasis [67,68]. In this study, we have demonstrated that miR-99b-5p expression can simultaneously target and inhibit the expression of *AR* and *MTOR*, leading to the suppression of EMT-mediated metastasis in AA PCa and EA CRPC. Recently, Zheng et al. showed that androgen stimulation induces EMT-mediated metastasis in AR-positive PCa, but not AR-negative PCa cells. SiRNA knockdown of eIF5A leads to upregulation of E-cadherin and downregulation of N-cadherin and Vimentin in AR-positive PCa (such as VCap and 22Rv1) [69]. In addition, a genomic study by Fletcher et al. has revealed that inhibition of miR-346, miR-361-3p, and miR-197 resulted in inhibition of the AR expression level, leading to suppression of the EMT event and EMT-mediated metastasis in AR-positive LNCaP and C4-2 cells [70]. Similar to these two studies, our study used four AR-positive PCa cell lines, LNCaP (metastatic EA PCa), C4-2B, and 22Rv1 (EA CRPC), and MDA PCa 2b (AA PCa), as our in vitro cell model to test the efficacies of miR-99b-5p alone and in combination with Enz or Abi in PCa. MiR-99b-5p mimic directly targets/inhibits the expression of AR, instead of modulating AR activity via AR antagonist, which may avoid inversely inducing/promoting EMT (i.e., in AR-negative PCa) and more effectively inhibit AR signaling and AR-mediated EMT in PCa, especially in AR-positive CRPC.

**Figure 8:**
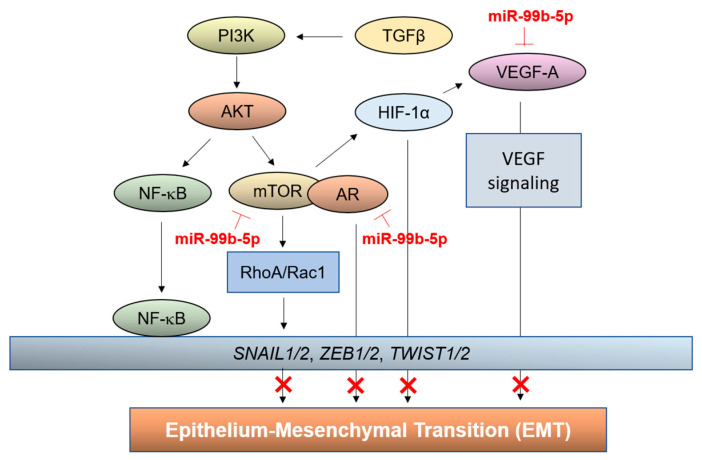
Graphical representation of miR-99b-5p-mediated suppression of EMT in AA PCa and CRPC. As illustrated, miR-99b-5p targets and inhibits the expression of *MTOR*, *AR*, and *VEGFA*, consequently suppressing the AR-, mTOR/RhoA/Rac1-, VEGF-, and HIF-1α-mediated activation of EMT.

An illustration representing the miR-99b-5p-mediated inhibition of EMT is presented in Figure 8. To date, this is the first study to demonstrate the involvement of miR-99b-5p in the regulation of EMT, through modulating AR, mTOR, HIF1α, and VEGF signaling.

## 5. Conclusions and Future Perspectives

In conclusion, this is the first study that demonstrates miR-99b-5p as a potent EMT-suppressive miRNA that inhibits EMT-mediated metastasis/angiogenesis, through simultaneously targeting *AR*/*MTOR* and downregulating mTOR, AR, HIF-1, and VEGF signaling in AR-positive PCa. Further elucidating the molecular mechanism of the miR-99b/mTOR/AR signaling axis in regulating EMT-mediated metastasis will warrant the development of novel therapeutic strategies for aggressive PCa, such as AA PCa and CRPC. Despite the promising potential for miR-99b-5p mimic in the inhibition of EMT in AA PCa and CRPC, challenges remain in clinical application. Further developing an efficient delivery system, generating a chemically stable miR-99b-5p mimic, and minimizing the off-target effect of miR-99b-5p will facilitate the development of this miRNA-based therapy for treating CRPC in preclinical and clinical settings.

## Data Availability

The data supporting the conclusions of this article will be made available by the authors on request.

## Supplementary Materials

The following supporting information can be downloaded at: www.mdpi.com, Full pictures of the Western blots.
