# Supplementary figures and images for: Combination of miR-99b-5p and Enzalutamide or Abiraterone Synergizes the Suppression of EMT-Mediated Metastasis in Prostate Cancer
Source: Cancers (Basel). 2024 May 19;16(10):1933. doi: 10.3390/cancers16101933 (PMC11119738; doi:10.3390/cancers16101933)

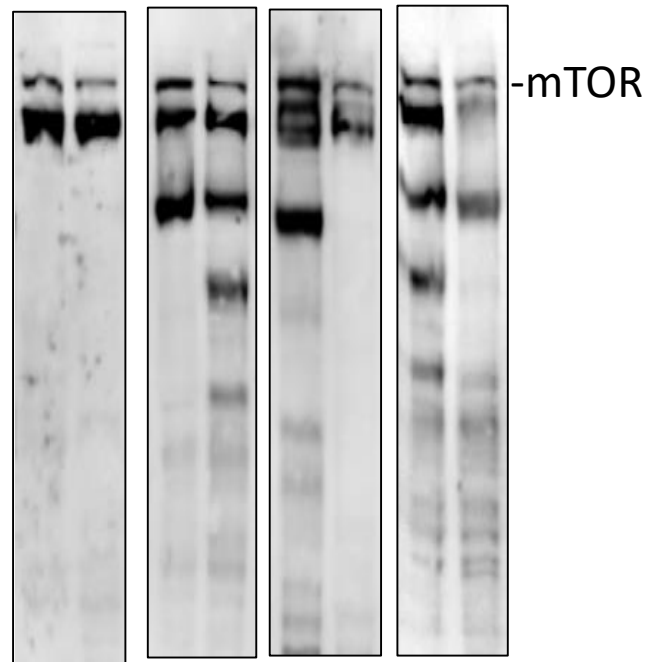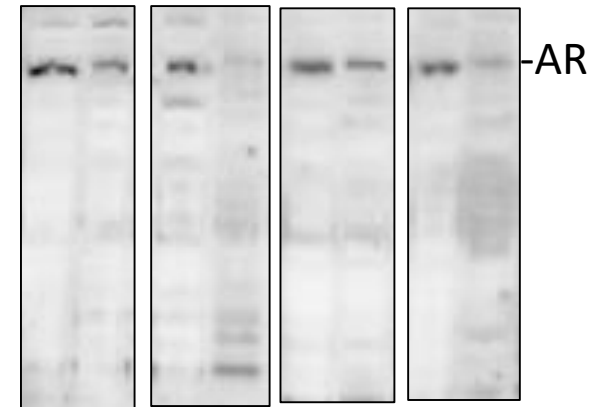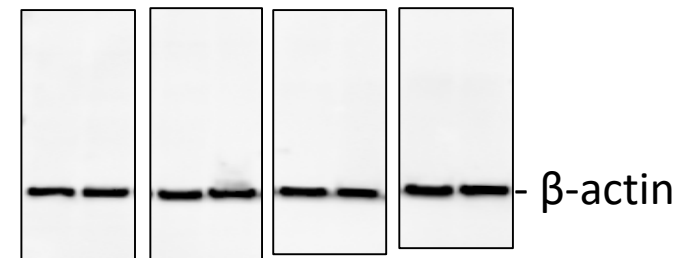

**Raw data for Figure 3A**

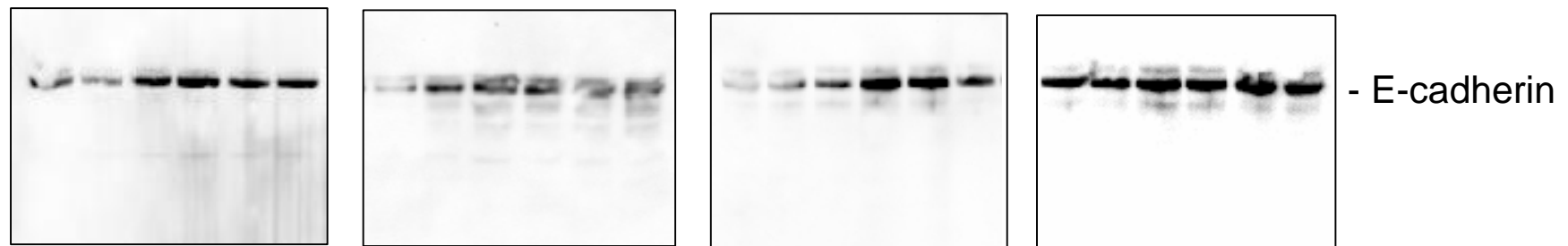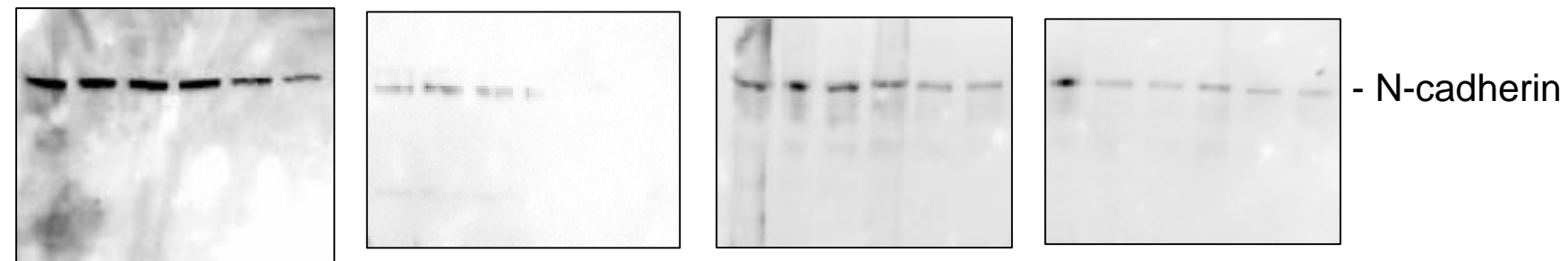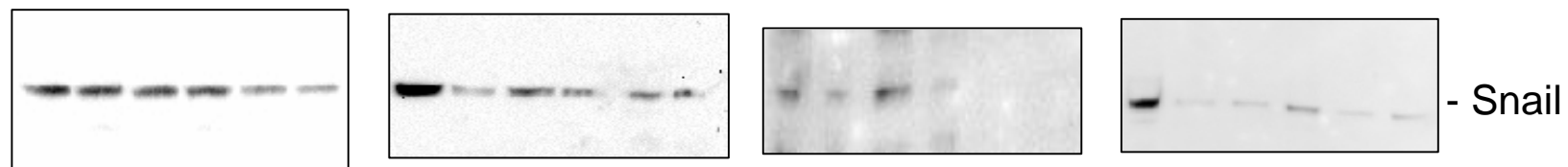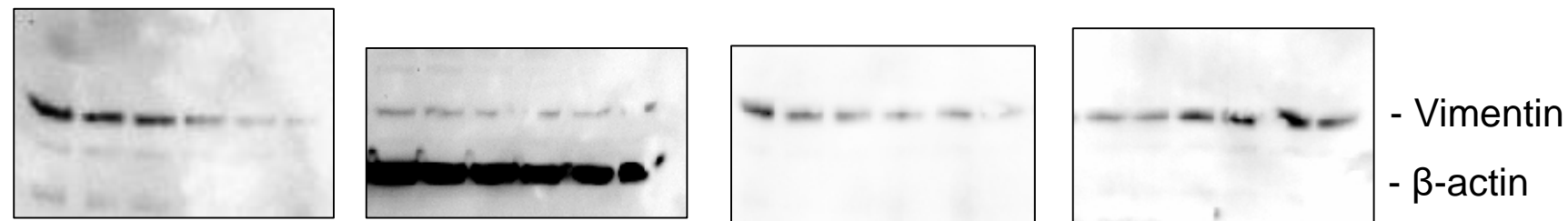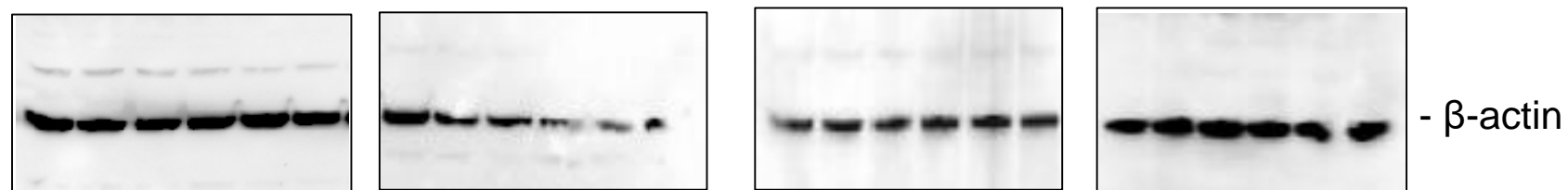

**LNCaP**

**C4-2B**

**22Rv1**

**MDA PCa 2b**

**Raw data for Figure 3A**

Supplement: Supplementary file 1 [file cancers-16-01933-s001.zip › cancers-2988832-supplementary.pdf]
